# Faunistics of Tiger Beetles (Coleoptera: Cicindelidae) from Pakistan

**DOI:** 10.1673/031.010.11601

**Published:** 2010-07-24

**Authors:** Muhammad Ather Rafi, Wiesner Jürgen, Muhammad Abdul Matin, Ahmed Zia, Amir Sultan, Falak Naz

**Affiliations:** ^1^ National Insect Museum, National Agricultural Research Centre, Islamabad-Pakistan,; ^2^ Dresdener Ring 11, D-38444 Wolfsburg, Germany

**Keywords:** distribution, biogeography, *Cylindera (Eriodera) albopunctata*, *Cicindela viridilabris*, *Neocollyris (Neocollyris) redtenbacheri*

## Abstract

The present biogeographic distribution of tiger beetle fauna is an attempt to register all modern taxa from Pakistan. It includes 55 taxa under 14 genera and 11 subgenera. Three species, *Cylindera (Eriodera) albopunctata* (Chaudoir 1852), *Cicindela viridilabris* (Chaudoir 1852) and *Neocollyris (Neocollyris) redtenbacheri* (Horn 1894) are recorded from Pakistan for the first time.

## Introduction

Biogeographically, the major part of Pakistan is Palaearctic (Hindu Kush, Karakorum, western Himalaya, Sulaiman Range, North Pakistan sandy desert and western Indus Valley) while the rest of the area is Oriental (Indus River Delta, eastern Indus Valley desert, Thar desert, Rann of Kutch in southern Punjab and eastern Himalaya) and traces of Afrotropical (Ethiopian region) from southern Iran to extreme southwestern of Baluchistan. The Hindu Kush, Karakorum, and Himalaya are a major biogeographic boundary between the subtropical and tropical flora and fauna of the Indian subcontinent and the temperate-climate Palaearctic ecozone. It is interesting to point out that the insect fauna, especially tiger beetles, completely confirm the transitional position of Pakistan between Palearctic and Oriental regions.

Tiger beetles (Cicindelidae) have been an appropriate indicator taxon for determining regional patterns of biodiversity (Pearson and Cassola 1992; Cassola and Pearson 2000), because the taxonomy of this group is stabilized, biology and general life history are well understood, they are readily observed and manipulated in the field and the family occurs world wide inhabiting many different habitat types. Each species tends to be specialized within a narrow habitat and the family includes species of potential economic importance (Pearson and Cassola 1992). In addition, diversity patterns of tiger beetles are often correlated with that of other groups (Pearson and Cassola 1992; Rodriguez et al. 1998) and there is much interest in these natural predators to control certain crop pests (Rodriguez et al. 1998).

Tiger beetles have worldwide distribution (except Tasmania, Antarctica and some remote oceanic Islands) that cover a variety of habitats ranging from alpine meadows to desert grasslands and tropical rain forests (Pearson 1988; Rodriguez et al. 1998). The total number of species presently known is over 2700 (Cassola Rome-Italy). Most of the species require habitats with access to bare ground, such as stream and pond edges, salt flats, dunes and open patches in grasslands (Pearson 1988; Hoback et al. 1998). Each species rarely occurs in more than one or a very few habitat types (Pearson 1984; Rodriguez et al. 1998).

In the Indian subcontinent literature on tiger beetles started with listing of species by Schaum 1863; Atkinson 1889 and Horn 1905a; 1905b. Annandale and Horn (1909) provided an annotated listing of the species found in the Indian Museum, Calcutta, accompanied by data on geographic distribution and habitats. Fowler (1912) compiled the first comprehensive list of all the genera of tiger beetles on the Indian subcontinent. Horn (1915) treated all the genera comprehensively on worldwide perspective. Dover and Ribeiro (1921, 1923); Horn (1926) and Heynes-Wood and Dover (1928) brought together much information on the synonymy, type depository, and geographic distribution of the species and subspecies. Horn (1938) provided a means of identifying species and subspecies using illustrations of elytral patterns and Rivalier (1950, 1958, 1961, 1971) developed a classification mainly using male genitalic characters. Mandl (1963) presented the results of expeditions of the Indus River in Kashmir and India.

Pearson and Ghorpade (1989) presented biogeographical information for tiger beetles of the Indian subcontinent. A comprehensive review on *Cicindela (sensu auctorum)* of the entire Indian subcontinent was provided by Acciavatti and Pearson (1989).

There have been very few publications on the Pakistan tiger beetles. Horn (1897), Fleutiaux (1899) and Maindron (1899) presented early collection records from Karachi. Fowler (1912), Chaudhry et al. (1966, 1970) provided survey results from the vicinities of Quetta, Kohat, Hazara and Swat. Cassola (1976) presented the results of collecting at Karachi and Lahore. Korell (1984) presented the biogeography data and notes on the morphology of some species collected from western and northern Pakistan. Recently Cassola and Wiesner (2009) reported a new species to science from Baluchistan. The present biogeographic distribution of tiger beetle fauna is first attempt to register all modern taxa of tiger beetles from Pakistan.

## Materials and Methods

The data were gathered through tiger beetle specimens housed at the National Insect Museum (NFM), National Agricultural Research Centre (NARC), Islamabad and specimens collected during northern area expedition in June of 2007. Furthermore, specimens that were housed at Pakistan Forest Institute (PFI, Peshawar) and Pakistan Natural History Museum (PMNH, Islamabad) were also examined. Many of the above mentioned museums collection specimens were sent to Fabio Cassola for identification and/or reconfirmation of already identified species.

### List of species

**Family: Cicindelidae Latreille 1802 Genus *Callytron* Gistl 1848**
***Callytron gyllenhalii* (Dejean 1825)**
  Remarks: Known from costal Pakistan: Sind: Karachi: Sandspit, 15 miles west Karachi (Cassola 1976; Acciavatti and Pearson 1989); According to Weisner (unpublished data) this species is also reported from India (Maharashtra).
  Bio-ecological Zone: Palaearctic.
***Callytron malabaricum*** (Fleutiaux and Maindron 1903)
  Remarks: Earlier this species was reported by Fleutiaux and Maindron (1903); Maindron and Fleutiaux (1905); Horn (1926) from India: Bombay (Malabar). Cassola (1976) from Pakistan: Baluchistan: Hab; 40 km West to Karachi. Acciavatti and Pearson (1989) from Sind and western coast of India: Maharashtra and Kerala.
  Bio-ecological Zone: Oriental.
***Callytron monalisa* (Horn 1927)**
  Remarks: Described from Iran (Horn 1927) but recently recorded from Pakistan as well (Shook and Wiesner 2006).
  Bio-ecological Zone: Palearctic.

**Genus *Calochroa* Hope 1838**
***Calochroa sexpunctata* (Fabricius 1775)**
  Material Examined: Pakistan: Sind:Karachi, 23. x. 2007, 1 ♂ leg. M. Atique Akhter. (det. and located with Wiesner).
  Remarks: Earlier reported by Fowler (1912) from India, Sri Lanka, Myanmar and China. Chaudhry (1966) reported from Bangladesh (Former East Pakistan). Chaudhry et al. (1970) collected it from Pakistan: NWFP: Hazara (Batgram) and Northern Areas: Gilgit. It is known from Indo-Malaysian region, Sri Lanka, India (Acciavatti and Pearson 1989), Thailand (Iacovone 2003), Vietnam (Dudko and Dubatolov 2000–2001). This species is also found in Sri Lanka, India (Tamil Nadu, Andhra Pradesh, Orissa, Western Bengal, Andaman Is, Nicobar Is), Central Nepal, Afghanistan, Pakistan and Philippines (Weisner, unpublished data).
  Bio-ecological Zone: Paleo-oriental.
***Calochroa bicolor atavus* (Horn 1920)**
  Remarks: Known from northwestern India (Uttar Pradesh, Himachal Pradesh, Punjab) and Pakistan (Acciavatti and Pearson 1989).
  Bio-ecological Zone: Oriental subspecies.
***Calochroa bicolor haemorrhoidalis* (Wiedemann 1823)**
  Material Examined: Pakistan: Punjab: Rawalpindi, 1–2. viii. 1984, 1 ♀, leg. Richter, (det. and located with Wiesner); NWFP: Bajor: Mohmand Agency, 20. v. 2008, leg. M. Iqbal (det. Wiesner 2009) ex NIM.
  Remarks: Earlier this subspecies was reported from Sri Lanka: Hambantota District, east central India: Madhya Pradesh, Orissa, Bihar, Rajasthan to southern India: Andhra Pradesh, Karnataka, Tamil Nadu and Pakistan: Punjab (Acciavatti and Pearson 1989; Werner and Wiesner 2008).
  Bio-ecological Zone: Oriental.
***Calochroa flavomaculata* (Hope 1831)**
  Remarks: Recorded from Pakistan by Cassola (2010). Generally occurring throughout the Indian subcontinent eastward into Southeast Asia and the Philippines (Acciavatti and Pearson 1989; Wiesner 1992). Recently reported from China: Yunnan Provinc (Wu and Shook 2007). According to Weisner (unpublished data) it is known from Pakistan, Nepal, India, Bangladesh, Andaman Is, Sri Lanka, Myanmar, Thailand, Vietnam, Laos, Cambodia, China (Guangdong, Hainan, Hong Kong, Sichuan, Xizang, Yunnan), Taiwan, Philippines.
  Bio-ecological Zone: Oriental.

**Genus *Calomera* Motschulsky 1862**
***Calomera angulata* (Fabricius 1798)**
  Remarks: Generally occurring throughout the Indian subcontinent eastward into Southeast Asia (Acciavatti and Pearson 1989). Known from India: Himachal Pradesh (Uniyal and Sivakumar 2007). Recently reported from China (Wu and Shook 2007). According to Weisner (unpublished data) it is known from Afghanistan, Pakistan (Margalla Hills), Nepal, Sikkim, India, Myanmar, Sri Lanka, Thailand, Laos, Cambodia, Vietnam, Malaysia (Malacca), Indonesia (Sumatra, Sumbawa, Borneo), Philippines (Luzon), Taiwan, China (Anhui, Fujian, Guangdong, Guizhou, Hainan, Hebei, Henan, Hubei, Jiangsu, Jiangxi, Shaanxi, Shanxi, Sichuan, Yunnan, Zheijang).
  Bio-ecological Zone: Oriental.
***Calomera aulica* (Dejean 1831)**
  Material Examined: Pakistan: Sind: Karachi, date unknown, T.R. Bell, 1 ♂*,* ex NIM; Sakrand, 25. iii. 2006, 1 ♀, leg. Falak Naz, ex NIM (det. F. Cassola, 2010); Karachi: Hawks Bay, 20. iii. 2007, 3 ♂♂, 2 ♀♀, leg. M. Ashraf, ex NIM (det. Rafi 2009); Sandspit, 21. iii. 2007, 1 ♂, 2 ♀♀ leg. Asad Ali, ex NIM (det. Rafi 2009); Umer Kot, 14. iii. 2007, 2 ♀♀, leg. Anjum Shazad, ex NIM (det. Cassola, 2010).
  Remarks: Reported by Maindron (1899) from Karachi: Kimarri. Cassola (1976) from Karachi: Sandspit; 20 km West to Karachi). Acciavatti and Pearson (1989) this species is widely distributed across northern Africa and the Middle East and entering Indian subcontinent along the southern Pakistan coast (Baluchistan and Sind. According to Weisner (unpublished data) this species found from Greece (S. Pelopones), Morocco, Algeria, Tunisia, Libya, Egypt, Sudan, Djibouti, Israel, Lebanon, Jordan, Syria, Saudi Arabia, Yemen, Bahrain, United Arab Emirates, Oman, Iraq, Somalia, Iran, Pakistan (Baluchistan, Sind, Karachi), Senegal, Gambia, Guinea Bissau (Cacheu, Oio), Guinea, Chad, Cape Verde, Angola, Kenya (North Eastern).
  Bio-ecological Zone: Palaearctic and Afrotropical species.
***Calomera chloris* (Hope 1831)**
  Material Examined: Pakistan: Northern Areas: (Gilgit airport) 07. x. 1959, leg. Unknown, ex NIM; Juglot, 15. iv. 2008, 1 ♀, leg. Anjum Shehzad (det. and located with Cassola 2010); Juglot, 19. vi. 2009, 5 ♂♂*,* 3 ♀♀, leg. Falak Naz, ex NIM (det. Rafi 2009) Punjab: Murree (Angoory), 13. vi. 2006, 1 ♂*,* leg. Mishkatullah, ex NIM (det. Rafi 2009); Multan: Chitter Watta, 01.v. 1954, 4 ♂♂*,* 4 ♀♀, leg. Student, ex NIM (det. and 1 ♂ with Cassola 2010); Chitter Watta, 1. v. 1954,1 ♂, leg. Sultan, ex NIM (det. Rafi 2009); Talagang (Pir Nara), 16. vi. 09, 1 ♀, 3 ♂♂, leg. A. Zia, ex NIM; NWFP: Swat (Karakar), 18. iv. 1964, 8 ♂♂, 4 ♀♀, leg. M. Ismail, ex PFI; Dir: Temergraha, 15. vii. 06, 1 ♀, leg. Amir Sultan, ex NIM (det. Rafi 2009); Charsada (Ser Deryab), 25. x. 2007, leg. Fida, ex PMNH (det. Rafi 2009); Peshawer: Warsak, 26. x. 2007, 9 ♂♂, 7 ♀♀, leg. Fida, ex PNHM (det. Rafi 2009); Bisham, 15–21. vi. 2008, 1 ♂, 2 ♀♀, leg. Anjum Shazad, ex NIM (det. 1 ♂ Cassola 2010); Sind: Bamboor (sea belt), 8. ix. 2008, 1 ♂, 1 ♀, leg. Akhter (det. and located with Wiesner).
  Remarks: Fowler (1912) reported this species from Kashmir, Northern Areas (Gilgit), Nepal and India; Chaudhry et al. (1966) from NWFP: Kohat; Toybanda near Kohat. Acciavatti and Pearson (1989) found across northern India (Anunachal Pradesh, Assam, West Bengal, Sikim, Bihar, Uttar Pradesh, Haryana, Punjab, Himachal Pradesh, Indian Jammu and Kashimr) Bhutan, Nepal and Pakistan (Northwest Frontier, Punjab). From India: Himachal Pardesh (Uniyal and Sivakumar 2007). According to Weisner (unpublished data) it is known from Afghanistan (Nengrahar), Pakistan (NWFP, Punjab), Nepal, Bhutan, India (Arunachal Pradesh, Assam, Western Bengal, Bihar, Uttar Pradesh, Haryana, Punjab, Himachal Pradesh, Jammu, Kashmir), Sikkim, Laos, ?Myanmar.
  Bio-ecological Zone: Paleo-oriental.
***Calomera diania* Tschitscherine 1903**
  Remarks: Known from Iran (Abusher, Dalaki, Borazjan), Iraq, Pakistan {Baluchistan (Turbat) and N.W.F.P}, Oman, Kuwait (Weisner, unpublished data).
  Bio-ecological Zone: Palaearctic.
***Calomera fischeri elongatosignata* (Horn 1922)**
  Material Examined: Pakistan: Baluchistan: Panjgur, 23. iii. 1962, 1 ♀, leg. S. M. Sher; ex NIM (det. Cassola 2010).
  Remarks: Reported from West Afghanistan (Mandl 1961), Persian Baluchistan (Mandl 1972), Pakistan: Baluchistan: Quetta: Hanna (Korell 1984), Turkmenistan and Kyrghyzstan (Dudko and Dubatolov 2000– 2001). According to Weisner (unpublished data) this species occurs in Iraq, Iran, Pakistan (Baluchistan), Afghanistan (Herat, Maimana, Nengrahar), Kyrgyzstan, Kazakhstan, Tadzhikistan, Turkmenistan, Uzbekistan, Oman, and United Arab Emirates.
  Bio-ecological Zone: Palaearctic.
***Calomera funerea assimilis* (Hope 1831)**
  Remarks: Reported from India (Pearson and Ghorpade 1989). According to Weisner (unpublished data) this subspecies also known from Pakistan, Nepal, India (Himachal Pradesh, western Bengal, Assam, Arunachal Pradesh, Uttar Pradesh, Meghalaya), Sikkim, Bhutan, Bangladesh, Myanmar, Thailand, Laos, China (Hainan, Sichuan, Yunnan).
  Bio-ecological Zone: Oriental.
***Calomera littoralis afghana* (Mandl 1955)**
  Remarks: Earlier Mandl (1955; 1981) reported this subspecies from Afghanistan: Kabul, Korell (1984) from Pakistan: Baluchistan: Quetta (Hanna).
  Bio-ecological Zone: Palaearctic.
***Calomera littoralis conjunctaepustulata* (Dokhtouroff 1887)**
  Remarks: Mandl (1981b; 1982b) reported this subspecies from India (Bombay). Reported from central portion of Palaearctic region including Pakistan: Baluchistan and Sind (Acciavatti and Pearson 1989), Karachi (Putchkov and Matalin 2003). Also known from Russia: west Siberia, Novosibirsk district, Ukraine Turkmenistan, Tadzhikistan, Kazakhstan, Europe (Dudko and Dubatolov 2000–2001; Putchkov and Matalin 2003).
  According to Weisner (unpublished data) this subspecies found in Iran (Baluchistan, Khuzistan, Nirisee), Azerbaijan, Georgia, Ukraine, Russia (S + C European Territory, west Siberia), Afghanistan (Herat, Kuschka), Kyrgyzstan, Pakistan (Baluchistan, Sind), India (Bombay, Dernah), NO Tibet, Tadzhikistan, Uzbekistan, Kazakhstan, Mongolia (Chovd aimak, Uvs aimak), China (Xinjiang, Xizang).
  Bio-ecological Zone: Palaearctic.
***Calomera plumigera macrograptina* (Acciavatti and Pearson 1989)**
  Remarks: Known from Pakistan: Punjab (Acciavatti and Pearson 1989). Also known from northern India: Punjab, Uttar Pradesh, Haryana, Orissa, Bihar, West Bengal, Assam; Nepal and Bangladesh (Acciavatti and Pearson 1989).
  Bio-ecological Zone: Oriental.

**Genus *Chaetodera* Jeannel 1946**
***Chaetodera albina* (Wiedemann 1819)**
  Remarks: Reported from Pakistan: Punjab; northern India: Punjab, Bihar, West Bengal, Orissa, Haryana, Utar Pradesh and Bangladesh: Rajshahi (Acciavatti and Pearson 1989) and also known from Nepal. (Weisner, unpublished data).
  Bio-ecological Zone: Oriental.
***Chaetodera vigintiguttata* (Herbst 1806)**
  Material Examined: Pakistan: NWFP: Peshawar, 5. ix. 1963. leg. Ayub. ex PFI (det. Zia 2009).
  Remarks: Known from Pakistan: Lahore: near River Ravi (Cassola 1976), India (Pajni and Bedi 1973, 1974; Uniyal and Sivakumar 2007). According to Weisner (unpublished data) it is known from Pakistan (Punjab, Lahore), India (Punjab, Uttar Pradesh, Haryana, Bihar, western Bengal, Assam, Sikkim, Orissa), Nepal (Bheri), Bhutan, Bangladesh (Dacca).
  Bio-ecological Zone: Oriental.

**Genus *Cicindela* Linneaus 1758 Subgenus *Cicindela* s. str.**
***Cicindela* (*Cicindela) granulata stoliczkana* Bates 1878**
  Remarks: Earlier reported by Heynes-Wood and Dover (1928) from India, Jammu and Kashmir, Jehlum Valley . Reported distribution by Acciavatti and Pearson (1989) from extreme northwest mountainous part of the Indian subcontinent in Pakistan (Punjab, NWFP). Known from China and Soviet central Asian republics: Tadjkistan; Kyrghyzstan; Kzakhstan; Uzbekistan (Acciavatti and Pearson 1989; Dudko and Dubatolov 2000–2001).
  Bio-ecological Zone: Palaearctic.

**Genus *Cosmodela* Rivalier 1961**
***Cosmodela intermedia* (Chaucoir 1852)**
  Material Examined: Pakistan: Punjab: Rawalpindi, 6. viii. 1982, 1 ♂, 4 ♀♀, leg. Richter, (det. and located with Wiesner 2009); Rawalpindi, 1–2. viii. 1984, 1 ♂, leg. Richter (det. and located with Wiesner); Islamabad, 6. vi. 2005, 1 ♂, 1 ♀, leg. Khalid, ex PMNH (det. Rafi 2009); Islamabad: 15. viii. 2006, 1 ♂, 1 ♀, leg. Khurrum, ex NIM (det. Rafi 2009); Islamabad: Margalla Hills, 25. vii. 2007, 1 ♀, leg. Amir Sultan, ex NIM (det. Rafi 2009); Islamabad: Simly Dam, 29. viii. 2008, 1 ♂, leg. A. Zia, ex NIM (det. Rafi 2009); Nowshera: Sodi village, 17. vi. 2009, 5 ♂♂, leg. A. Zia, ex NIM (det. Rafi 2009); NWFP: Bala Kot, 1. viii. 1963, 1 ♂, leg. S. M. Khan, ex PFI (det. Rafi 2009); Swat: Khawza-Khela, 21. viii. 1963, 1 ♂, leg. S. M. Khan; Hazara: Bhara ziarat: Doonga Gali, ex PFI (det. Rafi 2009); 26. vii. 1964, 4 ♂♂, 3 ♀♀, leg. M. Ismail, ex PFI; Kohistan: Kaghan: Balakat, 29. vi. 1977, 1 ♂, leg. de Freina, (det. and located with Wiesner); Abbottabad: Harno, 08. viii. 2008, 2 ♂♂, 1 ♀, leg. M. Ather, ex NIM (det. Rafi 2009); Abbottabad: Harno, 28. vii. 2008, 4 ♂♂, 2 ♀♀, leg. Amjad Bukhari, ex NIM (det. and 1 ♂ with Cassola 2010); Dadar, 14. vii. 2007, 1 ♂, leg. Zubair, ex NIM (det. Rafi 2009); Sind: Karachi: Sandspit, 9. viii. 1984, 1 ♀, leg. Richter (det. and located with Wiesner); Kashmir: Muzaffarabad: Guldana, 05. ix. 2007, 1 ♂, leg. Anjum Shazad, ex NIM (det. and located with Cassola 2010); Muzaffarabad: Shahdara, 08. viii. 2007, 2 ♂♂, 4 ♀♀, leg. Anjum Shazad, ex NIM (1 ♂ det. and located with Cassola 2010); 1 ♂, 4 ♀♀, ex NIM (det. Rafi 2009).
  Remarks: Known from Afghanistan through Pakistan (NWFP, Punjab) and northern India (Jammu and Kashmir, Punjab, Uttar Predesh) and Nepal. (Acciavatti and Pearson 1989). Also reported from India: Himachal Pardesh (Acciavatti and Pearson 1989; Bhargav et al. 2006; Uniyal and Sivakumar 2007). Also occurred in W + NC Nepal (Weisner, unpublished data).
  Bio-ecological Zone: Paleo-oriental.

**Genus *Cylindera* Westwood 1831 Subgenus *Cylindera* s.str.**
***Cylindera (Cylindera) obliquefasciata descendens* (Fischer 1825)**
  Material Examined: Pakistan: NWFP: Chitral: Madaglasht, 24–27. vi. 1983, 1 ♀, leg. Eckweiler (det. and locataed with Wiesner); Chitral: Madaglasht, 5–7. vii. 1982, 1 ♂, 1 ♀, leg. Erber and Heinz (det. and located with Wiesner); Northern Areas: Chilas, 11. vii. 1998, 1 ♂, 1 ♀, leg. E. G. Csorba and L. Ronkay (det. and located with Wiesner); Gilgit: Pander Lake, 11. vi. 2008, 1 ♂, 1 ♀, leg. Anjum Shehzad ex NIM (1 ♂ located with Cassola, det. Rafi 2009); Gopis: Khalti Lake, 11. vi. 2008, 1 ♂, 1 ♀, leg. Anjum Shazad, ex NIM (det. Rafi 2009); Skardu, 16. vi. 2008, 1 ♂, leg. Anjum Shazad, ex NIM (det. Rafi 2009).
  Remarks: Recorded recently from Pakistan by Cassola (2010). According to Weisner (unpublished data) it is also known from Tadzhikistan, Kyrgyzstan, Uzbekistan, Kazakhstan, Russia (west. Siberia), Turkmenistan, Mongolia, Afghanistan, Iran, Pakistan, India (Kashmir), China (Qinghai, Xinjiang, Zhejiang).
  Bio-ecological Zone: Palaearctic.

**Subgenus *Eriodera* Rivalier 1961**
***Cylindera (Eriodera) albopunctata* (Chaudoir 1852)**
  Material Examined: Pakistan: NWFP: Takht Bai, 7–9. vii. 1998, 1 ♂, 1 ♀, leg. G. Csorba and L. Ronkay (det. and located with Wiesner).
  Remarks: New to Pakistan. Known form Northern India: Punjab, Uttar Pradesh, Himachal Pradesh, West Bengal, Sikkim, Assam; Nepal and Bhutan (Acciavatti and Pearson 1989). Recently reported from China: Yunnan Province (Wu and Shook 2007).
  Bio-ecological Zone: Oriental.

**Subgenus *Eugrapha* Rivalier 1950**
***Cylindera (Eugrapha) agnata* (Fleutiaux 1890)**
  Material Examined: Pakistan: Baluchistan: Kuchh, 24. vi. 1964, leg. S. M. Khan, ex PFI; Mastang, 4. vii. 1964, leg. S. M. Khan, ex PFI.
  Remarks: Earlier reported from India: Bengal, Sikkam and Madras (Fowler 1912), Baluchistan: Quetta: Kuchh (Chaudhry et. al. 1966). Acciavatti and Pearson (1989) from India (Kerala, Tamil Nadu, Karnataka, Andra Pradesh, Orissa, West Bengal, Haryana and Punjab) into Pakistan (Baluchistan) and northern Myanmar (Burma).
  Bio-ecological Zone: Oriental.
***Cylindera (Eugrapha) bigemina* (Klug 1834)**
  Material Examined: Pakistan: Sind: Karachi: Malir, 20. vii. 1957, 2 ♂♂, 2 ♀♀, leg. Sultan ex NIM; Punjab: Rawalpindi (Khanna), 23. vii. 1963, 1 ♂, leg. Unknown, ex CABI, Rawalpindi; NWFP: Abbottabad, 22–27. vii. 1984, 1 ♂, 3 ♀♀ leg. Richter (det. and located with Wiesner); Harno (Abbottabad), 01-vii-2009, 1 ♂, leg. Zia, ex NIM, (det. Zia 2009).
  Remarks: Known from Pakistan: Punjab: Rawalpindi: Sohan river (Dover and Ribeiro 1921), Afghanistan and Nepal (Mandl 1967b, 1972a), Sind: Keenjhar lake: 120 Km East of Karachi (Cassola 1976), Pakistan (NWFP) and India (Jammu and Kashmir, Punjab, Uttar Pradesh, Madhya Pradesh, Bihar, west Bengal, Karnataka) and Nepal (Acciavatti and Pearson 1989), India: Himachal Pardesh; Uniyal and Sivakumar 2007).
  Bio-ecological Zone: Oriental.
***Cylindera (Eugrapha) brevis* (Horn 1905)**
  Material Examined: Pakistan: Sind: Karachi: Malir, 20. vii. 1957, 1♀, leg. Sultan (det. Cassola 2010); Hyderabad, Miani forest, 21. viii. 1959, 1 ♀, S. M. Din, ex NIM (det. by Cassola 2010); Punjab: Rawalpindi, 6. viii. 1982, 2 ♀♀, leg. Richter (det. and located with Wiesner); 1–2. viii. 1984; Rawalpindi, 2 ♂♂, 2 ♀♀, leg. Richter (det. Wiesner); Pir Nara (Talagung), 15-vii-2009, 1♂ 1♀, leg. Zia, ex NIM, (det. Zia 2009).
  Remarks: Known from Pakistan: Sind and Punjab and northern India: Punjab, Himachal Pradesh, Uttar Pardesh, Haryana and Bihar (Acciavatti and Pearson 1989), also known from Afghanistan (Weisner, unpublished data).
  Bio-ecological Zone: Oriental.
***Cylindera (Eugrapha) cognata* (Wiedemann 1823)**
  Remarks: Previously reported from India: Punjab: Chandigarh (Pajni and Bedi 1973), Pakistan: Lahore: River Ravi. (Cassola 1976), India (Tamil Nadu, Punjab, Haryana, Uttar Pradesh, Bihar, Andhra Pradesh, West Bengal, Orissa), Nepal and Bangladesh (Acciavatti and Pearson 1989).
  Bio-ecological Zone: Oriental.
***Cylindera* (*Eugrapha*) *grammophora* (Chaudoir 1852)**
  Material Examined: Pakistan: Punjab: Attock: 04. vi. 2009, 1♀, leg. Ramzan, ex NIM (det. Zia 2009).
  Remarks: Reported from India: Bangal, Punjab and Dehra Dun (Horn 1926), Chandigarh (Pajni and Bedi 1973), Himachal Pardesh (Uniyal and Sivakumar 2007), also known from Pakistan: Lahore: River Ravi (Cassola 1976), northern Pakistan, northern India, Nepal and Bangladesh. (Acciavatti and Pearson 1989).
  Bio-ecological Zone: Oriental.
***Cylindera (Eugrapha) sublacerata* (Solsky, 1874)**
  Material Examined: Pakistan: Northern Areas: Gilgit, 31. vii. 1987 (det. and located with Wiesner); Goopi: Khalti Lake, 11. vi. 2008, 1 ♀, leg. Anjum Shazad, ex NIM (det. Cassola 2010); Singal (Ghizer), 18-vi-2009, 1 ♂, leg. Naz, ex NIM (det. Zia 2009). Remarks: Earlier reported by Mandl (1961) from Pakistan. Known throughout the southern republics of the Soviet Union (Turkmenstan, Uzbekistan, Tadjishikistan, Kirghisatan), and Adjoining portion of Iran, Afghanistan, China, northern Pakistan and India Jammu and Kashmir (Acciavatti and Pearson 1989), Known from Kazakhistan, Tadjihikistan, Turkmenistan (Dudko and Dubatolov 2000–2001; Franzen and Gebert 2004).
  Bio-ecological Zone: Palaearctic.
***Cylindera (Eugrapha) sublacerata balucha* (Bates 1878)**
  Material Examined: Pakistan: Baluchistan: Quetta: Hanna, 25–27. v. 1983, 1 ♂, leg. Eckweile (det. and located with Wiesner).
  Remarks: Known from Pakistan: Baluchistan and Skardu (Korell 1984), adjoining parts of Iran and Afghanistan (Acciavatti and Pearson 1989).
  Bio-ecological Zone: Palaearctic.
***Cylindera* (*Eugrapha*) *venosa* (Kollar 1836)**
  Material Examined: Pakistan: Punjab: Ghambeer Bridge (Talagung), 15. vii. 2009, 1 ♂1 ♀, leg. Zia, ex NIM (det. Zia 2009).
  Remarks: Known from Kashmir and Sind: Karachi (Dover and Ribeiro 1923; Horn 1926). Punjab: Pakistan: Rawalpindi: River Sohan (Mandl 1963), Lahore: Ravi River (Cassola 1976), except peninsular India, Sri Lanka and Nepal it occurs throughout Indian subcontinent eastward into Southeast Asia (Acciavatti and Pearson 1989), Myanmar (Wiesner 2006), India: Himachal Pradesh (Uniyal and Sivakumar 2007). During present study specimen was also examined from Bangladash: Dhanjuri Dinajpur: Dist. Mapelli, 1963, 1♀. east NIM (det. Cassola 2010) also known from Pakistan (Lahore), India (west Bengal, Uttar Pradesh, Haryana, Punjab, Assam, Arunachal Pradesh, Meghalaya, Gujarat), Sikkim, Bangladesh, Thailand, Cambodia (Weisner, unpublished data).
  Bio-ecological Zone: Oriental.
***Cylindera (Eugrapha) mesoepisternalis* (Horn 1934)**
  Remarks: This species was described from Skardu (Horn 1934).
  Bio-ecological Zone: Palaearctic

**Subgenus *Ifasina* Jeannel 1946**
***Cylindera (Ifasina) decempunctata* (Dejean 1825)**
  Material Examined: Pakistan: Punjab: Khewra, 01. viii. 2006, 1 ♂, leg. Fida, ex PMNH (det. Cassola 2010); Islamabad: NARC, 16. vii. 2007, 1 ♀, leg. A. Zia, (det. and located with Cassola 2010). Northern Areas: Gilgit: Naltar, 6. vii. 2008, 1 ♂, leg. A. Akhter (det. and located with Wiesner). NWFP: Mansehra, 14. viii. 1963, M. Ismail, 1 ♂, ex PFI, Peshawer; Mansehra, 22. vii. 1964, G. U. Chaudhry, 1 ♂, ex PFI, Peshawer (det. by Cassola 2010); Hazara: 20. iv. 1967. leg. Nasrullah, ex PFI, Peshawer.
  Remarks: Reported from Bengal, Burma (Myanmar), Tonkin and Combodia (Fowler 1912). Pakistan. NWFP: Hazara: Mansehra (Chaudhry et al. 1970), Pakistan: Punjab and northern India: Uttar Pardesh, Punjab, Haryana, Bihar, West Bengal, Assam; Nepal and Bangladesh: Dacca; east ward into Barma (Acciavatti and Pearson 1989), also known from Myanmar, Thailand, Laos, Vietnam, Cambodia (Weisner, unpublished data).
  Bio-ecological Zone: Oriental.
***Cylindera (Ifasina) subtilesignata* (Mandl 1970)**
  Material Examined: Pakistan: NWFP: Donga galli, 22. viii. 1964, leg. M. Ismail, 1 ♀, ex NIM (det. Cassola 2010); Abbottabad: Harno, 01. vii. 2009, leg. Ashraf, 1 ♀, ex NIM. (det. Wiesner 2009).
  Remarks: Recorded recently from Pakistan by Cassola (2010). Previously known from Nepal and northern India (Punjab, Uttar Pradesh, Himachal Pradesh, West Bengal) and Burma. Acciavatti and Pearson 1989), also known from India (Meghalaya, Sikkim), Myanmar, Malaysia (Malacca) (Weisner, unpublished data).
  Bio-ecological Zone: Oriental.
***Cicindela viridilabris* (Chaudoir 1852)**
  Material Examined: Pakistan: NWFP:Parachinar, 18.vii.1964, leg. Cheema. ex PFI; Azad Kashmir: Muzafarabad, 10.vii.09, 1 ♂, leg. Amjad, ex NIM (det. Rafi 2009).
  Remarks: Known from northern India (Haryana, Punjab, Himachal Pradesh, Uttar Pradesh, Madhya Pradesh, Orissa, Bihar) and Nepal (Acciavatti and Pearson 1989). Also recorded by Chaudhary et al. (1966) from Bangladesh (Former East Pakistan).
  Bio-ecological Zone: Paleo-oriental.
Genus *Grammognatha* Motschulsky 1850
***Grammognatha euphratica* (Dejean 1822)**
Material Examined: Pakistan: Kashmir: Muzaffarabad, 22. ix. 1959, 1 ♂, leg. Ghori, ex NIM (det. Cassola 2010); Sind: Karachi, 01. vi. 2006, 1 ♀, leg. Zubair; Mithi: Thar Desert, ex NIM (det. Cassola 2010); 20. ix. 2008, 1 ♀, leg. Akhter (det. Wiesner); Mirpurkhas, 9. vi. 2008, 3 ♂♂, 1 ♀, leg. Akhter (det. Wiesner).
  Remarks: Known from Turkey, Syria, Saudi Arabia (Franzen 2001). According to Wiesner (unpublished report) this species occurred in Spain (Almeria, Murcia, Alicante), Morocco (Moulouya, Melilla, Outatel Haj, Foum Zguid, Mhamid), Algeria, Tunisia, Libya, Senegal, Greece (Rhodes, Crete), Cyprus, Turkey, Egypt, Israel, Jordan, Lebanon, Syria, Iraq, Yemen, Kuwait, Saudi Arabia, United Arab Emirates, Oman, Djibouti, Iran, Pakistan (Karachi).
  Bio-ecological Zone: Palaearctic.

**Genus: *Hypaetha* Leconte 1860**
***Hypaetha copulata* (Schmidt-Goebel 1846)**
  Material Examined: Pakistan: Sind: Karachi (Sandspit, 20 km West to Karachi), 8–10. viii. 1982, 4 ♂♂, 5 ♀♀, leg., Richter (det. and located with Wiesner); Karachi: Sandspit, 9. viii. 1984, 2 ♂♂, 2 ♀♀, leg. Richter (det. and located with Wiesner).
  Remarks: Known from Karachi (Maindron 1899) and Arab Emirates (Wiesner 1996; Hellyer and Aspinalls 2005), According to Weisner (unpublished data) it is also known from Iran (Bushire), Pakistan (Sind, Karachi), Saudi Arabia, Oman.
  Bio-ecological Zone: Palaearctic.
***Hypaetha ornatipennis* (Schilder 1953)**
  Remarks: Known from coastal Pakistan: Sind and Iran (Acciavatti and Pearson 1989).
  Bio-ecological Zone: Palaearctic.
***Hypaetha quadrilineata* (Fabricius 1781)**
  Material Examined: Pakistan: Sind: Karachi: Sandspit, 9. viii. 1984, 1 ♂, leg. Richter (det. and located with Wiesner).
  Remarks: Earlier, Fowler (1912) reported it from sandy area of Manorah near Karachi, Cassola (1976) reported this species from Sind: Karachi: Sandspit. Naviaux (1983) reported it from Iran (Naviaux 1987) from the Malacca peninsula. Acciavatti and Pearson (1989) reported this species from the entire coast of the Indian subcontinent and the coast of Sri Lanka, Myanmar (Barma) and Malay Peninsula, and considered *millingeni* Bates, 1878 to be merely an individual variation. Also known from Thailand (Wiesner 1992).
  Bio-ecological Zone: Oriental.

**Genus *Lophyra* Motschulsky 1859 Subgenus *Lophyra* Motschulsky 1859**
***Lophyra (Lophyra) cancellata intemperata* (Acciavatti and Pearson 1989)**
  Material Examined: Pakistan: Sind: Karachi: Malir, 7. v. 1951, 1 ♀, leg. Nawaz, ex NIM (det. Cassola 2010); Malir, 30. iii. 1959, 1 ♂, leg. Yasin, ex NIM (det. Cassola 2010); Karachi: Malir, 30. viii. 1964, 1 ♂, leg. Latif, ex NIM (det. Rafi 2009); Karachi: Malir, 25. vii. 1964, 1 ♀, leg. Latif ex NIM (det. Rafi 2009); Islam Kot, 27. iii. 2008, 2 ♂♂, leg. Ishaq Mastoi, ex NIM (det. Cassola 2010); Punjab: Kundian, 10. v. 1960, 1 ♀, leg. Unknown, ex PFI (det. Cassola 2010).
  Remarks: According to Acciavatti and Pearson (1989) this subspecies occurs from Pakistan (Sind and Punjab) across northern India (Andhra Pradesh, Assam, Bihar, Gujarat, Haryana, Madhya Pradesh, Orissa, Uttar Pradesh, Sikkam, West Bengal) and Thailand. Also known from India (Arunachal Pradesh, Meghalaya) and Nepal (Weisner, unpublished data).
  Bio-ecological Zone: Oriental.
***Lophyra (Lophyra) catena* (Fabricius 1775)**
  Remarks: Acciavatti and Pearson (1989) reported from Pakistan (Sind), India (Rajasthan, Punjab) generally through southern and eastern India and Sri Lanka, Wiesner (2006) from Myanmar.
  Bio-ecological Zone: Oriental.
***Lophyra (Lophyra) histrio* (Tschitschérine 1903)**
  Material Examined: Pakistan: Punjab: Lyallpur (Faisalabad), 2. viii. 1948, 1 ♀, leg. Student, ex NIM (det. Cassola 2010); Sind: Usta Mohd, 16. iii. 1950, 1 ♂, leg. Osmani, ex NIM (det. Cassola 2010); Malir, 7. v. 1951, 2 ♀♀, leg. Nawaz, ex NIM (det. Cassola 2010); Upper Sind: Jamrau head, 14. ix. 1953, 2 ♂♂, leg. S. M. Din, ex NIM (det. Cassola 2010); Karachi: Malir, 17. ix. 1958, 1 ♀, leg. Aziz, ex NIM (det. Cassola 2010); Karachi: Malir, 7. x. 1958, 1 ♂, 1 ♀, leg. M. Yasin, ex NIM (det. Cassola 2010), Karachi: Malir, 19. x. 1959, 1 ♀ Aziz, ex NIM (det. Cassola 2010); Karachi, 25. vii. 1964, 1 ♂, leg. Latif, ex NIM (det. Cassola 2010); Karachi, 30. viii. 1964, 1 ♀, leg. Latif, ex NIM (det. Cassola 2010); Karachi: Malir: Goth Memon, 20. viii. 1964, 1 ♂, leg Latif, ex NIM (det. Cassola 2010).
  Remarks: Known from Iran and Afghanistan (Mandl 1961 and 1967a), Pakistan: Karachi. (Horn 1903) and Cassola (1976) from Sind: Thatta (120 km East of Karachi). Naviaux (1983) reported this species from Iran. Acciavatti and Pearson (1989) reported from India (Rajistan) and Pakistan (Sind) westward into Afghanistan and Iran. Also known from Saudi Arabia, United Arab Emirates, Oman (Weisner, unpublished data).
  Bio-ecological Zone: Palaearctic.

**Subgenus: *Spilodia* Rivalier 1961**
***Lophyra* (*Spilodia) vittigera* (Dejean 1825)**
  Material Examined: Pakistan: D.I.Khan: Rang pur, 18. vi. 2009, 1 ♂, 1 ♀, leg. Zubair, ex NIM (det. Rafi 2009).
  Remarks: Horn (1926) reported this species from Bangladesh and India (Dehra Dun). Pajni and Bedi (1973) from India (Chandigarh), Cassola (1976) from Pakistani Punjab: Lahore (Ravi river). Second author reported this species from Pakistan (Punjab, Lahore), India (Punjab, Haryana, Uttar Pradesh, Bihar, Western Bengal), Nepal, Bangladesh.
  Bio-ecological Zone: Oriental.

**Genus: *Myriochila* Motschulsky 1857 Subgenus: *Monelica* Rivalier 1950**
***Myriochila (Monelica) akhteri*Cassola and Wiesner 2009**
  Remarks: Recently described (Cassola and Wiesner 2009) from Lowralai, Baluchistan.
  Bio-ecological Zone: Endemic species of a mainly Palaearctic subgenus.
***Myriochila (Monelica) fastidiosa* (Dejean 1825)**
  Remarks: Fowler (1912) reported this species from Punjab: Rawalpindi, Kashmir; India: Sikkim, Assam, Bengal, Madras and central India; Ceylon (Sri Lanka) and Burma (Myanmar). Chaudhry et al. (1970) reported this species from NWFP: Kohat (Toybanda). Acciavatti and Pearson (1989) reported that this species was widely distributed throughout Pakistan; India and Sri Lanka to Myanmar (Burma).
  Bio-ecological Zone: Oriental
***Myriochila (Monelica) fastidiosa litigiosa* (Dejean 1825)**
  Remarks: Known from Pakistan (Jammu), India (Himachal Pradesh, Punjab, Haryana, Uttar Pradesh), Nepal (Weisner, unpublished data).
  Bio-ecological Zone: Oriental
***Myriochila (Monelica) leucoloma* (Chaudoir 1852)**
  Material Examined: Pakistan: Punjab: Lyallpur, 2. viii. 1948, leg. Student, 2 ♂♂, ex NIM (det. Cassola 2010).
  Remarks: Previously known from India and Nepal (Acciavatti and Pearson 1989). Recently reported from Pakistan Cassola (2010).
  Bio-ecological Zone: Oriental.

**Subgenus: *Myriochila* Motschulsky 1862**
***Myriochila (Myriochila) dubia* (Horn 1892)**
  Material Examined: Pakistan: Sind: Islam Kot, 25–27.iii.2008, 1♂, 2♀♀, leg. Ishaq Mastoi, ex NIM, (det. and 1♀ with Cassola 2010).
  Remarks: New to Pakistan. Earlier this species was reported by Heynes-Wood and Dover (1928) from Myanmar (Burma). Acciavatti and Pearson (1989) from India (Asam, Nagaland) and Thailand.
  Bio-ecological Zone: Oriental.
***Myriochila (Myriochila) melancholica* (Fabricius 1798)**
  Material Examined: Pakistan: Sind: Islam Kot, 25.iii.2008; Punjab: Bhakar, 15.v.2008, 2♂♂, 6♀♀, leg. A. Akhter; (det. and located with Wiesner).
  Remarks: Known from Portugal (Algarve), Spain; Malta; France; Italy; Greece; Cyprus; Turkey; Morocco; Algeria; Gambia; Tunisia; Libya; Egypt; Israel; Saudi Arabia; Yemen; Bahrain; United Arab Emirates; Oman; Iran; Syria; Iraq; E. Ciscaucasia; Caucasus Major; Armenia; Pakistan; Afghanistan; Nepal and India (Wiesner 2001). According to Weisner this species is present in Portugal (Algarve), Spain (Almeria, Alicante, Murcia, Granada, Gerona, Balearic Is, (Ibiza, Mallorca, Menorca), Malta, France (Corsica), Italy (Sardegna, Sicily, Calabria, Latium), Albania, Greece (Thessalia, Moree, Attica, Eubee, Acarnanie, Peloponnes, Crete, Cephalonia, Rhodes), Cyprus, Turkey (Anatolia), Morocco, Algeria, Tunisia, Libya, Egypt, Israel, Jordan, Lebanon, Saudi Arabia, Yemen, Kuwait, Bahrain, United Arab Emirates, Oman, Iran, Syria, Iraq, Azerbaijan, Georgia, Kazakhstan, Armenia, Kyrgyzstan, Tadzikistan, Turkmenistan, Uzbekistan, Pakistan (Sind, Karachi), Afghanistan (Herat, Shibargan, Paktia, Nengrahar), Nepal, India (Maharashtra, Rajasthan, Punjab, Haryana, Uttar Pradesh, Madhya Pradesh, Bihar, Western Bengal), China (Xinjiang), Cape Verde Is, Senegal, Gambia, Benin, Sierra Leone, Guinea Bissau, Guinea, Chad, Ivory Coast, Togo, Ghana, Nigeria, Cameroon, Sao Tomé and Principe, Equatorial Guinea, Central African Republic (Yalinga), Congo (Brazzaville), Democratic Republic of Congo (Shaba), Tanzania, Kenya, Somalia, Ethiopia, Sudan, Zimbabwe, Malawi, Mozambique, Burkina Faso, Angola, Namibia, Botswana, Zambia, Swaziland, South Africa (Cape Province, Venda, Limpopo, KwaZulu-Natal), Madagascar, Seychelles.
  Bio-ecological Zone: Paleo-oriental and Afrotropical.
***Myriochila (Myriochila) undulata* (Dejean 1825)**
  Material Examined: Pakistan: NWFP: Kalam, 16. ix. 1963, leg. M. Khan, ex PFI, Peshawer; Punjab: Sakasar, 27. viii. 2007, 1 ♂, leg. Amir Sultan, ex NIM (det. Cassola 2010).
  Remarks: Earlier known from India, and Hong Kong (Fowler 1912), NWFP: Kalam, near river (Chaudhry et al. 1966), throughout Indian subcontinent (Acciavatti and Pearson 1989), India (Himachal Pardesh; Uniyal and Sivakumar 2007), Nepal, Pakistan, India, Sri Lanka and Bangladesh (Werner and Wiesner 2008).
  Bio-ecological Zone: Oriental.

**Genus: *Rhytidophaena* Bates 1891**
***Rhytidophaena limbata* (Wiedemann 1823)**
  Material Examined: Pakistan: Punjab: Islamabad, 23. vii. 2006, 1 ♀, leg. M. Ather, ex NIM, (det. Rafi 2009); Toba Take Singh, 17. vii. 2007, 1 ♀, leg. Zubair, ex NIM (det. Cassola 2010).
  Remarks: Firstly recorded from Pakistan by Fowler (1912). Known from Nepal, Bangladesh, India (Meghalaya, Assam) Pakistan: Punjab (Weisner, unpublished data).
  Bio-ecological Zone: Oriental.

**Table 1.**
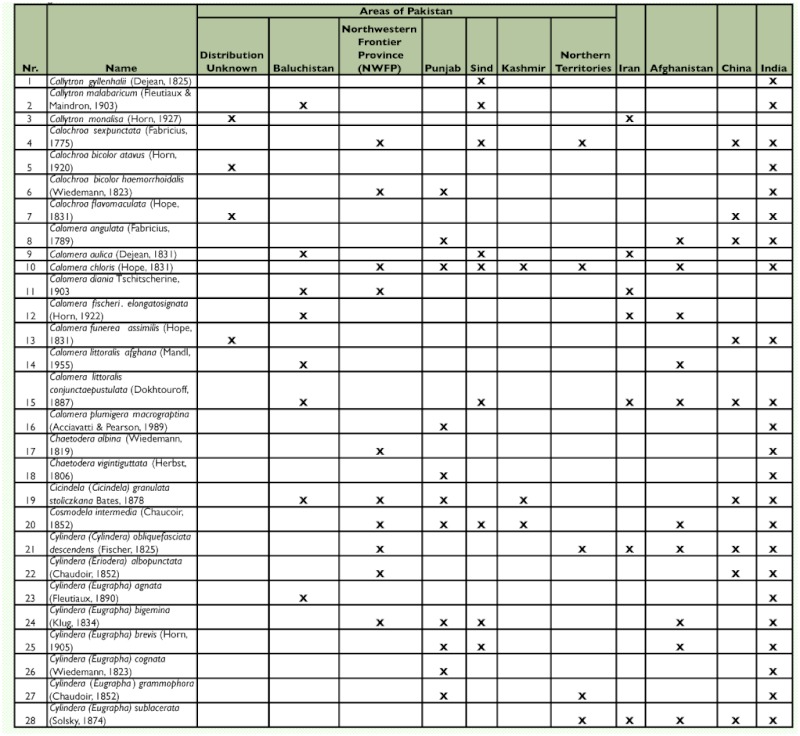
Tiger beetle fauna of Pakistan

**Table:**
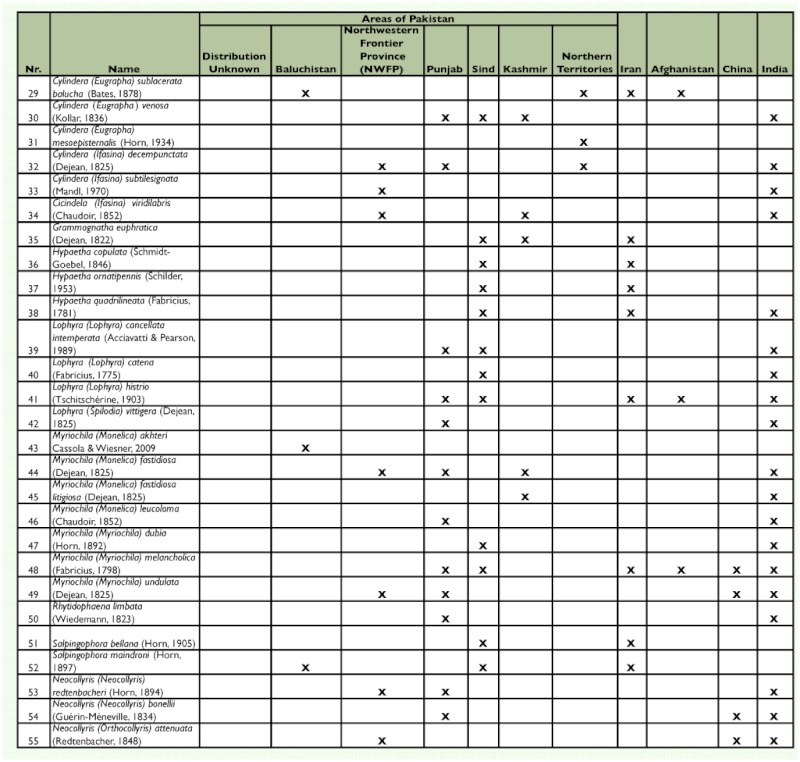

**Genus: *Salpingophora* Rivalier 1950**
***Salpingophora bellana* (Horn 1905)**
  Remarks: Known from Pakistan: Sind: Karachi westward along the Persian Gulf of Iran and Kuwait. (Acciavatti and Pearson 1989) also known from Iraq, Saudi Arabia (Weisner, unpublished data)
  Bio-ecological Zone: Palaearctic.
***Salpingophora maindroni* (Horn 1897)**
  Material Examined: Pakistan: Sind: Karachi, date (unknown), 1 ♂, leg., T. R. Bell, ex NIM; Khairpur, 9. vii. 1963, leg. M. Ismail, ex PFI, Peshawar; Mirpurkhas, 9. vi. 2008, 2 ♀♀, leg. Akhter (det. Wiesner); Baluchistan: Somiani: Lasbella, 23. i. 1951, 1 ♀, ex NIM.
  Remarks: Horn (1897); Mindron (1899) reported this species from Karachi (Kimari); Dover and Ribeiro (1923) Baluchistan; Rivalier (1950) from Iran (Bushire), Karachi; Cassola (1976) Karachi (Sandspit, 20 km West to Karachi); Thatta (120 km East to Karachi). Known from Pakistan: Baluchistan and Sind (Acciavatti and Pearson 1989).
  Bio-ecological Zone: Palaearctic.

**Genus *Neocollyris* Horn 1901 Subgenus: *Neocollyris* Horn 1901**
***Neocollyris (Neocollyris) redtenbacheri* (Horn 1894)**
  Material Examined: Pakistan: NWFP: Abbottabad, 22. vii. 1969, 1 ♂, leg. Unknown, ex NIM (det. Cassola 2010); Abbottabad (Harno), 28. vii. 2008, 1 ♂, leg. Amjad Bukhari, ex NIM (det. Cassola 2010); Punjab: Islamabad (Rawal Lake), 17. vii. 2008, 1 ♀, leg. Asad Ali, (det. and located with Cassola 2010); Islamabad (Margalla Hills), 31. vii. 2006, 2 ♀♀, leg. Amir Sultan, ex NIM (det. and 1 ♀ located with Cassola 2010).
  Remarks: New record for Pakistan. Earlier reported by Naviaux (1995) from Himalayan mountains. According to Weisner (unpublished data) this species occurred in Bhutan, Nepal, India (Assam, Meghalaya, Calcutta, Punjab).
  Bio-ecological Zone: Oriental.
***Neocollyris (Neocollyris) bonellii* (Guérin-Méneville 1834)**
  Material Examined: Pakistan: Punjab: Islamabad: Margalla Hills, 31. vii. 2006, 1 ♀, leg. Amir Sultan ex NIM (det. Cassola 2010); Islamabad: Margalla Hills, 12. viii. 2008, 2 ♀♀, leg. Asad, ex NIM (det. Rafi 2009).
  Remarks: Reported by Cassola (2010) from Pakistan (Islamabad). Known from Indonesia (Jawa, Bali, Sumatra, S. Utara, S. Barat, Bengkulu, Jambi, S. Selatan), Sumbawa, Sumba, Flores, Borneo (Kalimantan), Sulawesi (S. Utara, S. Tengah, S. Selatan)), Malaysia (Malacca), Singapore, Nepal, India, Bangladesh, Myanmar, Thailand, Laos, Cambodia, Vietnam, China (Fujian, Guangdong, Guangxi, Hainan, Hong Kong, Hunan, Yunnan, Zhejiang) (Weisner, unpublished data).
  Bio-ecological Zone: Oriental

**Subgenus: *Orthocollyris* Naviaux 1995**
***Neocollyris (Orthocollyris) attenuata* (Redtenbacher 1848)**
  Material Examined: Pakistan: NWFP: Swat: Mingora, 11. viii. 1963, 2♂ 1 ♀, leg. S. M. Khan, ex PFI (det. Rafi 2009); Bala Kot, 27. vii. 1964, 1 ♀, leg M. Ismail, ex PFI (det. Rafi 2009); Abbottabad, 21. vii. 1969, leg. M. Ismail, ex PFI (det. Rafi 2009); Doonga Gali, 22. vii. 1964, 2♀♀, leg. M. Ismail, ex PFI (det. Zia 2009); Punjab: Ghora Gali, 20. vii. 1962, 5 ♀♀, leg. M. Ismail, ex PFI (det. Rafi 2009).
  Remarks: Fowler (1912) reported this species from India (Punjab, Sikkam and Assam). Chaudhry et al. (1966) reported from NWFP: (Mingora, Balakot) and Bangladesh (former East Pakistan), Chittagong (Datmara). Reported from India (Simla, Darjeeling, Meghalaya, Arunachal Pradesh, Bengal, Punjab), Nepal, Myanmar, Bhutan, ?China: Xizang (Weisner, unpublished data).
  Bio-ecological Zone: Oriental.

## Discussion

These results indicate that the tiger beetle fauna of Pakistan includes 50 taxa in 14 genera and 11 subgenera (Table 1).

The biogeographic distribution of three taxa belonging to genus *Callytron* (*C. gyllenhalii* (Dejean) and *C. monalisa* (Horn)) is Palaearctic and Oriental for *C. malabaricum* (Fleutiaux and Maindron). *Calochroa bicolor atavus* (Horn) and *C. flavomaculata* occurs in Pakistan with Oriental biogeography. Under the genus *Calomera* Motschulsky six species were recorded from Pakistan, which include *C. angulata* Fabricius, *C. aulica* (Dejean), *C. chloris* (Hope), *C. diania* Tschitschérine, *C*. *littoralis* Fabricius, and *C*. *plumigera* (Horn).

The genus *Chaetodera* Jeannel is represented by two species, *Ch. albina* (Wiedemann) and *Ch. vigintiguttata* (Herbst), which reportedly have an Oriental distribution. Just one taxon in subgenus *Cicindela* s. str. represent the genus *Cicindela* Linneaus 1758 i.e. the Palearctic species *Cicindela (Cicindela) granulata* Gebler ssp. *stoliczkana* Bates. One single species, *C. intermedia* represents the Oriental genus *Cosmodela* in Pakistan, because the record of *C. didyma* (Chaudhry et al. 1966) is probably due to a labeling mistake (Cassola 2010).

Twelve species represent the genus *Cylindera* (in four subgenera). Their distribution is mostly Oriental [such as those of *C. (Eugrapha) agnata* (Fleutiaux), *C. (E) bigemina* (Klug), *C. (E) brevis* (Horn), *C. (E) grammophora* (Chaudoir), *C. (E) venosa* (Kollar), *C. (Eriodera) albopunctata* (Chaudoir), *C. (Ifasina) decempunctata* (Dejean), and *C.(I) subtilesignata* (Mandl)], with just three species being Palaearctic [*C. (C) obliquefasciata* (Adams), *C. (E.) sublacerata* (Solsky), and C*. (E) mesoepisternalis* (Horn)].

The genus *Grammognatha* Motschulsky is represented by the typonominal subspecies of *Grammognatha euphratica* (Dejean) having Paleo-oriental distribution. [Reported distribution of *G. euphratica* Latreille and Dejean and *G.(E) armenica* Laporte was Palaearctic (Kryzhanovskij et al. 1995; Cassola 1999; Franzen 2001; Iacovone 2003; Löbl and Smetana 2003; Anichtchenko and Chibilov 2005; Avgin and Özdikmen 2007)].

The genus *Hypaetha* Le Conte has presently three species: *H. copulata* (Schmidt-Goebel), *H. ornatipennis* (Schilder) and *H. quadrilineata* (Fabricius) the first two having a Palaearctic distribution (Maindron 1899, Cassola 1976; Acciavatti and Pearson 1989) and the last one being Oriental (Acciavatti and Pearson 1989).

*Lophyra (Lophyra) cancellata intemperata* (Acciavatti and Pearson) and *Lophyra (Lophyra) catena catena* (Fabricius) both belong to Oriental species arriving westwards to Pakistan (Acciavatti and Pearson 1989), while *L. (L.) histrio* (Tschitschérine 1903) is a Palaearctic species. *Lophyra* (*Spilodia) vittigera* (Dejean) is also basically Oriental and belongs to an Oriental subgenus (Horn 1926; Pajni and Bedi 1973; Cassola 1976).

Six species represent the genus *Myriochila* Motschulsky in Pakistan, namely three of the typonominal subgenus [*M. (Myriochila) melancholica* (Fabricius), *M. (M.) dubia* (Horn) and *M. (M.) undulata* (Dejean) and three of subgenus *Monelica* [*M. (Monelica) aktheri* Cassola and Wiesner 2009, *M. (M.) fastidiosa* (Dejean) and *M. (M.) leucoloma* (Chaudoir). *M. (Myriochila) melancholica* is perhaps the commonest and most widespread species in the genus, occurring in the Palearctic region, in the whole of Africa and in middle Orient eastwards to Pakistan.

Moreover, there are in Pakistan one species belonging to Himalayan genus *Rhytidophaena* Bates, *Rh. limbata* (Wiedemann), two species of the Palaearctic genus *Salpingophora* Rivalier. *S. bellana* (Horn) and *S. maindroni* (Horn) and possibly three more species of the Oriental genus *Neocollyris* Horn, which reportedly occur in lowland Himalayan areas (“Piémonts hymalayens”: Naviaux 1995).

## Conclusion

These results appear to support the hypothesis advanced by Pearson and Ghorpade (1989) that the tiger beetles fauna on the subcontinent is largely the result of numerous independent contributions from the Ethiopian, the Palaearctic and the Oriental faunas.
